# Harm reduction techniques among cisgender gay, bisexual, and queer men using anabolic androgenic steroids: a qualitative study

**DOI:** 10.1186/s12954-024-01121-8

**Published:** 2024-11-11

**Authors:** Eric Kutscher, Arslaan Arshed, Richard E. Greene, Mat Kladney

**Affiliations:** 1 Icahn School of Medicine at Mount Sinai, New York, NY USA; 2 NYU Grossman School of Medicine, New York, NY USA; 3 Bellevue Hospital Center, New York, NY USA

**Keywords:** Anabolic androgenic steroids, Steroids, LGBTQ, Gay, Bisexual, Queer, Sexual minority, Community based research

## Abstract

**Background:**

Anabolic androgenic steroids (AAS) are synthetic forms of testosterone frequently used as performance enhancing drugs among gay, bisexual, and queer (GBQ) men. Despite widespread use, associated harms, and the likely existence of an AAS use disorder, there is no medical consensus on standards of care for people who use AAS, with most medical providers focusing exclusively on abstinence. Individuals using AAS have developed community-based harm reduction strategies to mitigate these harms.

**Methods:**

This paper is a sub-analysis of qualitative data obtained through semi-structured interviews with GBQ men using AAS for 8 or more weeks recruited through convenience and snowball sampling from clinical sites and LGBTQ + venues in New York City as well as through social media. Interviews were coded with themes developed using reflexive thematic analysis. Data related to harm reduction techniques were then re-analyzed through a prevention strategies framework lens of primary, secondary, and tertiary harm prevention.

**Results:**

Thematic saturation was reached at twelve interviews in the primary analysis, with men reporting frequent use of multiple harm reduction techniques. For primary prevention, men avoided oral steroids and simultaneous substance use, tried to obtain AAS from reputable sources, used “cycling” to dose steroids, and practiced sterile injection techniques. Secondary prevention methods included patient-directed lab testing for hematocrit, liver and kidney function, cholesterol, prostate specific antigen, testosterone, and self-performed blood pressure checks. Tertiary prevention included donating blood and the use of medications without a prescription, including aromatase inhibitors, selective estrogen receptor blockers, aspirin, statins, angiotensin receptor blockers, clomiphene, and human chorionic gonadotropin.

**Conclusions:**

Despite many GBQ men experiencing harms from anabolic androgenic steroids, community members have often sought harm reduction techniques in lieu of abstinence. Though many of these techniques embrace clinical reasoning and may be more broadly applicable, additional research is needed to understand the impact of each intervention on the overall health of individuals using AAS.

## Background

Anabolic androgenic steroids (AAS) are synthetic versions of testosterone used as performance-enhancing drugs by millions of Americans [1]. AAS use often enhances secondary male sex characteristics, leading to increased muscle tone, strength, and endurance [2]. Though historically these effects were desired by individuals engaging in competitive sports and bodybuilding, AAS use is also seen among cisgender gay, bisexual, and queer (GBQ) men for various reasons, including social capital, adult film, escort work, self-medication for body dysmorphia, or to treat secondary hypogonadism from chronic AAS use [2–5]. Unlike transmasculine individuals, these cisgender men use nonprescribed AAS without a medical indication and often at higher doses to target supraphysiologic testosterone levels.

The addictive nature of AAS is gaining increasing recognition, with multiple studies identifying an “AAS use disorder” characterized by pharmacologic tolerance, inability to stop using when desired, and continued use despite harm [1, 6–9]. The health implications and physical harms related to AAS use continue to require further research [10, 11]. AAS use has been associated with higher risk of mortality [12], hypertension and liver abnormalities [13], cardiomyopathies, coronary artery disease, stroke, hepatitis, erythrocytosis, prostate growth, male pattern balding, and acne. Rates of hepatitis B, C, and HIV are higher among individuals who use AAS, with a disproportionately higher prevalence among gay men using AAS largely attributable to an increased risk of sexual exposure to HIV [14]. Overall, LGBTQIA + males using AAS have significantly higher rates of AAS associated health conditions compared to heterosexual peers.^15^

When seeking medical care, people who use AAS report encountering providers with limited experience managing AAS-related concerns, often with a primary focus on promoting abstinence [16, 17]. Providers themselves often feel inadequately prepared to care for patients using AAS [17], likely exacerbated by the lack of consensus on medical management for patients using AAS [18].

Given this context, many people who use AAS report a preference for obtaining medical information through online and peer support services [19]. Within this community, harm reduction practices are often discussed and shared, with some medical professionals advocating for a harm reduction approach to AAS use [16, 19–22]. However, the harm reduction strategies employed by GBQ men who use AAS have yet to be fully explored.

Sexual minority men who use AAS have been shown to be less likely than their heterosexual counterparts to seek help from a general practitioner [15]. Therefore, understanding this community’s particular methods of harm reduction is essential. This paper presents a secondary analysis of our qualitative study on AAS use among GBQ men [9] aiming to further explore the harm reduction techniques participants reported, and help develop a framework for healthcare providers when counseling GBQ men using AAS.

## Methods

We report on a sub-analysis of qualitative study using self-administered questionnaires and semi-structured interviews including cisgender adult men who identified as gay, bisexual, or queer and had used AAS for at least 8 consecutive weeks. Individuals with active psychotic or manic symptoms, exclusive use of prescription AAS by a provider with use only as prescribed, or a history of past or current prostate cancer were excluded from the study. Participants were recruited through a convenience sample from the Bellevue Hospital Center Adult Primary Care Clinic and social media postings. Additional participants were recruited by snowball sampling from already recruited participants until thematic saturation was reached. Interviews were recorded and transcribed using a computer-based transcription system (Otter.ai), and then analyzed using a reflexive thematic analysis approach. Complete methods and results for the study have been published elsewhere [9]. This study was approved by the NYU Langone Health and Bellevue Hospital Center Institutional Review Boards.

After completion of thematic analysis, authors extracted harm reduction related content from interviews for a post-hoc secondary analysis. Authors used a hybrid inductive-deductive method, first reviewing interviews for additional themes and then viewing these themes into the public health prevention framework [23–26]. The public health prevention framework was selected for framing of the harm reduction themes given its widespread use in preventive medicine and ability to account for biopsychosocial aspects of person-centered care.

## Results

From November 2021 through May 2023, twelve participants completed semi-structured interviews until thematic saturation was reached, with a cohort predominantly consisting of White, gay-identifying men with most completing a college degree or higher level of education. All participants (12 of 12) reported complications from steroid use, and seven of the twelve men met criteria for muscle dysmorphia using the Muscle Dysmorphia Disorder Index, though all had symptoms predating steroid use. Full participant demographics and associated harms are reported elsewhere [9].

Participants strongly felt that there were rational justifications for steroid use and described abstinence-based approaches as moralizing and insufficient:

It's very hard to convince them [doctors] that no, this [testosterone] is something that helps me. Please understand that I'm going to do them anyway—let’s work with this. It’s not helpful for someone to just tell you, they're bad stop. That's absolutely not helpful. It's it should be a discussion, not a directive (J, 49yo gay White man).

Thus, in applying themes from the interviews to the prevention strategies framework, a focus on preventing AAS use was not included. Instead, participants often emphasized efforts to prevent AAS-related harms from occurring through careful planning and research (primary prevention), increased monitoring for harms while using steroids (secondary prevention), and specific strategies to reduce AAS-induced harms when they occurred (tertiary prevention). These three sub-themes are outlined below and summarized in Table 1.

**Table 1:** Harm reduction techniques for primary, secondary, and tertiary prevention

Prevention strategy framework	Harm reduction techniques
Primary prevention: *planning and research to avoid AAS-related harm*	- Researching and information gathering - Cycling steroids - Hiring of coaches and personal trainers - Monitoring for purity and quality of AAS - Optimizing diet and cardiovascular exercise - Avoiding use of other substances - Sterile injection techniques - Utilization of Pre-Exposure Prophylaxis for HIV
Secondary prevention: *monitoring for AAS-related harms*	- Checking for testicular shrinkage, increased breast mass - Monitoring blood pressure and vital signs - Performing self-guided blood testing (hemoglobin/hematocrit, basic metabolic panel, liver function tests, lipid panel, testosterone levels)
Tertiary prevention: *reducing and treating AAS-related harms*	- Purchasing medications without prescription (off-label use of statins, aspirin, milk thistle, angiotensin receptor blockers, aromatase inhibitors) - Donating blood - Utilizing post-cycle therapy (off-label use of clomiphene, human chorionic gonadotropin, tamoxifen)

### 1. Preventing harm (primary prevention).

Knowledge and resource gathering were integral to the primary prevention of AAS-related harms. Multiple participants interviewed believed that steroid-related harm could be avoided through careful pre-use planning. Very few interviewees started using AAS without first conducting significant amounts of research. “I definitely wanted to become as familiar as I possibly could with all the different kinds of, like, avenues of damage. So, I could know what I could do to mitigate that as much as possible.” (G, 25yo gay White Hispanic man).

Patients reported taking extensive amounts of time to organize and create a steroid regimen that would be most likely to optimize their goals and least likely to result in secondary hypogonadism—a known side effect of AAS use. This included the use of “cycles” instead of continuous use, planning cycles in conjunction with other people using steroids and/or with internet guidance, and choosing steroids believed to be less toxic.

I was extraordinarily precise at measuring out the dosage and planning ahead for what my cycle would be and making sure that I have time off, like at least equivalent to the amount of time that I had been on cycle… Very strict about that. (J, 49yo gay White man)

To optimize efficacy and safety, many participants discussed using a coach, personal trainer, or peer as a harm reduction counselor. These individuals often claimed to help develop safer regimens and ensured maximized benefit with minimized harm. Coaches frequently recommended avoiding oral steroids due to their known hepatotoxicity.

Participants also expressed concerns about the purity and quality of the AAS, fearing that injecting contaminants could be dangerous. Multiple men reported sourcing steroids from friends or acquaintances, while others preferred to buy AAS from websites they believed to be more reputable. One individual purchased AAS internationally and brought them back to the United States. Prior to use, one participant always examined labels for signs of “homemade” steroids, such as home-printed labels or spelling mistakes.

Other methods of sourcing to optimize safety were reported. Multiple men discussed finding clinics where doctors would prescribe testosterone for non-medically indicated use, particularly citing online clinics as a means to easily obtain prescription testosterone. These clinics were seen as a way to ensure purity and obtain a safe supply of testosterone.

Aside from AAS planning, participants sought to optimize their health to prevent harm. Dietary changes were common. One individual shared “I’m constantly drinking water… like you need a gallon of water a day at minimum… and if you’re not religious about that, you will very much have a problem with your kidneys.” (J, 60yo gay White man). Others focused on maintaining a low-fat diet, sharing that if “you have too many cheat meals or you’re just sloppy, you can get fat accumulation around your organs, not just your midsection, which is of course very dangerous. (J, 39yo gay White man). Cardiovascular exercise was also thought to help decrease steroid-associated cholesterol risks.

Participants also felt that it was essential to avoid the concomitant use of other drugs and alcohol while using AAS. “If I know I’m taking a drug that is taxing on my liver, then I will make sure that I don’t drink during that period of time. No alcohol because I know that’s gonna exacerbate the problem.” (J, 49yo gay White man) Another participant reflected:

So a few people at the gym had suggested that I… lower it if I was going to be partying a lot. And I think the reason it makes some sense because… what I’ve noticed when I’m on it is I definitely need to eat more. And I definitely need to get plenty of fluids. And if I’m partying I’m not eating enough and I’m not getting enough fluids. So I’m leaving my body in a kind of depleted state… it just leaves me susceptible. It, like, lowers my immune system.

Lastly, all participants reported that they were HIV-negative and took precautions to prevent HIV and other infectious diseases. Men often noted that they had many sexual partners and engaged in unprotected anal sex, with some involved in adult film production. However, they widely used pre-exposure prophylaxis to prevent HIV infection (PrEP). All participants decreased the risk of acquiring blood-borne illness through injection practices, as they used new syringes for each injection and avoided sharing syringes. Some purchased syringes used for B12 injections from pharmacies or online, while others obtained needles from syringe exchange programs.

I do know places where a lot of gay men live, where a large portion, like a substantial portion of people who do make use of the needle exchange are using steroids and exchanging needles (J, 49yo gay White man).

### 2. Monitoring for harm (secondary prevention).

For secondary prevention, participants often felt that active surveillance was a form of prevention, allowing them to adjust their AAS practices if abnormalities were detected before more serious conditions developed. One individual summarized the importance of self-monitoring:

The way I sort of figured in my head is I'm not really doing any harm by doing this, like, physically, if it turned out that, you know, if it was something like, I'd been a pack a day smoker for years, and I found out that I had lung cancer and I'd better stop right now. Or if you know, I was had a terrible diet, and I found out I was pre diabetic and I’d have to stop right now. I think if I was told you're heading for liver failure, you got to stop right now or you're gonna die, you're gonna have to be on dialysis for the rest of your life, then I think I would stop. But I try to keep in top of what's going on with my body. I see my doctor regularly. I get blood work regularly and so far I've been okay, so if it ain't broke, right, and it seems to be better than not broke, it's actually seems to be working. (J, 49yo gay White man)

Screening for physical harms occurred after research on possible side effects, including checking for decreased testicular size, increased breast tissue, or monitoring vital signs and blood pressure. One man reflected, “I’ve been watching for those things…. I’ve researched and read about them all so I keep an eye out for those things and watch for it” (C, 37yo gay White man).

Self-guided blood testing was extremely common. Almost all participants mentioned that their main harm reduction technique was to “keep up on labs, you know, pretty routinely” (C, 37yo gay White man). “I’m very diligent about my blood testing,” another one shared (46yo Bisexual White man).

Specifically, participants wanted to check “hematocrit and iron [to] make sure my bloods aren’t getting too thick.. cholesterol—that’s an expensive test—so I check that once a year.” (64yo bisexual White man). Another focused on the comprehensive metabolic panel, sharing “Liver values are also a huge concern AST, ALT… Kidney function, protein that appears in the kidneys” (J, 39yo gay White man). One participant mentioned, “I take my blood glucose every morning” (K, 30yo gay Black man). Blood work was frequently performed at labs, paid for out of pocket, and obtained without a doctor’s order or insurance coverage.

### 3. Reducing harms (tertiary prevention).

In our cohort, individuals reported a variety of harms they attributed to AAS, ranging from aesthetic issues like gynecomastia, acne, and balding, to life-threatening conditions such as pneumonia, atrial fibrillation, and hypertrophic cardiomyopathy.

To address these concerns, most participants focused on harm reduction rather than abstinence from steroids. As one individual stated:

You know, you're pumping hormones into your body. So, there's going to be effects from it. It's, it's those that are more responsible, that understand what the effects are and know how to manage them or minimize them. (N, 49yo gay White man)

Multiple participants discussed instances of cellulitis from poor injection techniques when first starting AAS, which ultimately led to improvements in their sterile injection practices.

Elevated blood count (polycythemia) was a frequently reported side effect, with three patients reporting blood donation as a treatment method. One participant noted that donating blood “freaked him out” because “as you know, gay men can’t donate blood” (J, 60yo gay White man) but felt that it was the best harm reduction option.

A commonly reported phenomenon was the purchasing of medications without prescriptions to offset harm from steroid use. For some, this included the use of statins to decrease cholesterol. Liver supplements, including milk thistle, were mentioned by two participants as methods to help with hepatotoxicity. Daily aspirin was also taken by multiple participants to treat blood that was “too thick” (64yo bisexual White man).

Two participants reported purchasing non-prescribed angiotensin receptor blockers (ARB) for their “good effects for actually reversing left ventricular hypertrophy” (G, 25yo gay White Hispanic man). One of these participants reported that a specific ARB, telmisartan, was the most likely to improve AAS-induced cardiomyopathy.

To address hormonal side effects such as gynecomastia and night sweats, some participants used aromatase inhibitors.

I know my symptoms when my testosterone is either getting too high or my estrogen is too high… my sleep quality goes down or my erection quality goes down. It’s then I will usually use aromatase inhibitors to kind of purge the body of excess estrogen. (K, 30yo gay Black man)

When experiencing secondary hypogonadism, participants reported various methods to increase endogenous testosterone after a cycle, often called “post-cycle therapy.” One individual explained that:

What I do is what we call PCT—post cycle therapy. So I take a couple of different things that help trick my brain into thinking it’s not producing testosterone so it starts up again (N, 49yo gay White man).

Two types of post cycle therapy (PCT) were reported. Most commonly used were selective estrogen receptor modulators (SERMs) like clomiphene or tamoxifen, thought to promote luteinizing hormone and follicle stimulating hormone from the hypothalamus. Use of human chorionic gonadotropin (HCG) was also described, thought to act as an analog to luteinizing hormone directly on the testicles.

Clomid tends to make your body naturally start producing more testosterone as well, but also more sperm. And it kind of like gently brings your balls back to working mode… there’s HCG which is a subcutaneous injectable… it basically tricks your body into producing testosterone, no matter what (E, 39yo gay White Hispanic man).

The development of harms related to AAS use was universal amongst our participants. Due to perceived stigma and lack of knowledge in medical settings, many turned to their own communities for information and treatment rather than seeking help from healthcare providers. One participant reflected, “I don’t know how much the mainstream medical system really cares to deal with us” (G, 25yo gay White Hispanic man).

## Discussion

Despite every participant reporting harm from AAS use, all continued to use anabolic androgenic steroids. Many felt that abstinence-focused medical care was not aligned with their own goals, alienated them from medical settings, and further distanced them from seeking professional help. Similarly to individuals using other substances, conversations centered on abstinence were interpreted as stigmatizing and as barriers to receiving important medical information. Consequently, for this cohort, primary prevention was defined as avoiding complications from AAS use rather than abstaining from AAS use altogether.^24^

Though primary prevention of AAS-related harms was highly desired, participants reported having few reliable resources to rely upon. Many reported spending months or even years researching AAS, different modalities of use, and associated risks and benefits, searching both academic and lay literature for any information available. The value of online peer forums and coaches as facilitators of harm reduction information is similar in our cohort as seen in other studies [27, 28]. Men emphasized the lack of research and data that objectively outlined the safest ways to use AAS and minimize medical risk. Participants also expressed fascination with learning how to bypass hormonal regulations in the body but were frustrated that many medical providers and trusted medical resources were less knowledgeable about the nuances of AAS than they needed to be [29]. Further research in the field on AAS-related risks and risk-mitigation techniques was strongly desired.

Given the perception of inadequate data on AAS use and limited provider knowledge, many in our cohort performed screening for AAS-related harms outside of the medical system. Participants felt that self-ordered lab tests and vital sign monitoring allowed them access to information about their health without feeling judged and stigmatized by healthcare providers. Importantly, they used secondary prevention screenings not to decide to stop using AAS, but to intervene and adapt use as soon as necessary. Thus, the abstinence messaging of many providers alienated participants from seeking expert opinion and advice on what harms to screen for, both related to and not related to AAS use. Currently there is a dearth of guidelines on what screenings should be performed for individuals using AAS. Developing such a framework could help both patients and providers feel more aligned in their common goal of harm reduction.

When complications did arise, participants attempted to address these medical issues without professional guidance. Polypharmacy was common among participants, with many choosing not to disclose the use of most non-prescribed medications to providers (both AAS and other non-prescribed medications). By not disclosing these medications to providers, participants faced the risk of unanticipated drug interactions and increased side effects. Participants emphasized that disclosure was only possible if they knew there would be no documentation in the medical record due to the illegality of AAS use, and their fear of medicolegal consequences from disclosure. Additionally, the safety and efficacy of off-label use of certain medications, including post-cycle therapy, remains unknown and would benefit from further investigation.^30^

Like harm reduction methods for other substances, harm reduction for AAS use included the goal of preventing infectious disease. Given the relatively infrequent nature of AAS injections (typically once a week) and lack of acute withdrawal symptoms experienced from other drugs, men felt that they were able to plan in advance to obtain the materials they needed for a cycle. Participants denied sharing needles and did not perceive this as a common practice among individuals using steroids, in contrast to findings of other studies [14]. Though some reported connecting with syringe services programs, services at traditional harm reduction spaces are not usually tailored to the needs of people using AAS [31]. A few participants developed cellulitis at the injection site when they first started using AAS due to a lack of knowledge about sterile injection techniques, as found in other studies [32]. However, these participants later modified their practices to ensure greater safety.

With regards to HIV prevention, participants injected AAS intramuscularly and reported consistent use of new and sterile syringes. Participants denied any increased risk of blood borne diseases from injection practices, unlike as reported previously [14]. Instead, men in the study perceived higher rates of HIV among GBQ men using AAS to be result of increased rates of condomless anal sex. Men reported that enhanced masculine features would attract more sexual partners and heightened their own arousal and interest in sexual activity. However, despite high levels of unprotected sexual activity among participants [33], HIV risk was perceived as low in our cohort due to high PrEP adherence that often predated the use of AAS.

Significant resources were required by participants to practice harm reduction approaches to AAS use, with many men reporting spending hundreds of dollars per cycle. The need for resources is reflected in the final cohort of our study which was our cohort was predominantly White, college-educated gay men despite recruitment primarily from a public hospital clinic.

## Limitations

We had significant difficulty recruiting for our study, likely due to the stigma surrounding both sexual orientation and substance use. The final cohort of participants had limited socioeconomic diversity—it is unknown if this cohort reflects the demographics of the GBQ population using AAS given the significant cost and knowledge that participants relied on to use AAS. The requirement of a minimum of eight weeks of AAS use may also have biased our study to focus on individuals less likely to pursue abstinence and more interested in harm reduction approaches to AAS use. The participants in the study were all cisgender men, and additional research on the harm reduction techniques of transmasculine individuals self-medicating for gender affirming hormonal therapy requires additional investigation.

## Conclusions

Individuals using AAS often avoid mainstream healthcare due to stigmatization and the perception that providers lack the necessary knowledge to care for this population. GBQ individuals using AAS have developed and utilized harm reduction techniques to decrease the risk of AAS-related harms, monitor for complications from AAS use, and self-medicate for AAS-related complications. The safety and efficacy of these interventions are unknown and require additional research. Future studies should investigate the acceptability and feasibility of harm reduction counseling by healthcare providers, particularly among GBQ men, and with particular attention to individuals with increased socioeconomic diversity. Further development of best practices for harm reduction approaches to AAS use may help both patients and providers have a framework to discuss the care needs of people who use AAS.

## Data Availability

This work was approved by the Institutional Review Boards of NYU Langone Health and Bellevue Hospital Center, and all participants consented to partake in the study. Study consent forms prohibited the release of publicly available full participant transcripts but are available from the corresponding author on reasonable request.

## Abbreviations

AAS: Anabolic androgenic steroids
GBQ: Gay, bisexual and queer
LGBTQ +: Lesbian, gay, bisexual, transgender, queer, and other sexual and gender minorities
HIV: Human immunodeficiency virus
PrEP: Pre-exposure prophylaxis for HIV
PCT: Post cycle therapy
